# Geriatric nutritional risk Index predicts adverse outcomes across cardiovascular–kidney–metabolic syndrome: discovery in NHANES and external validation in CKM stage 4 patients undergoing PCI

**DOI:** 10.3389/fcvm.2026.1878842

**Published:** 2026-07-07

**Authors:** Hongyan Zhu, Jing Yang, Yuqi Fang, Yizhu Yan

**Affiliations:** Department of Cardiology, Beijing Luhe Hospital Affiliated to Capital Medical University, Beijing, China

**Keywords:** cardiovascular–kidney–metabolic syndrome, external validation, geriatric nutritional risk index, major adverse cardiovascular events, nutritional status, percutaneous coronary intervention, risk prediction

## Abstract

**Background:**

Cardiovascular–kidney–metabolic (CKM) syndrome imposes a high burden of adverse events, yet prognostic biomarkers remain scarce. We evaluated the prognostic value of the Geriatric Nutritional Risk Index (GNRI) across CKM stages 1–4 and in high-risk patients undergoing percutaneous coronary intervention (PCI).

**Methods:**

This discovery–validation study utilized 16,074 adults with CKM syndrome from NHANES (1999–2018) and an external validation cohort of 2,401 CKM Stage 4 patients undergoing PCI. Primary outcomes were all-cause mortality (discovery) and major adverse cardiovascular events (MACE; validation).

**Results:**

In the discovery cohort, compared with the highest GNRI quartile (Q4), Q1 was associated with significantly higher hazards of all-cause (HR 1.58; 95% CI 1.32–1.89) and cardiovascular mortality (HR 2.13; 95% CI 1.55–2.93); each 1-unit increase in GNRI was associated with a lower hazard of all-cause mortality (HR 0.95; 95% CI 0.94–0.97). In the validation cohort, Q1 vs. Q4 conferred a markedly increased risk of MACE (HR 2.52; 95% CI 2.03–3.13), with each 1-unit increase in GNRI associated with a lower risk of MACE (HR 0.95; 95% CI 0.94–0.96). Restricted cubic splines revealed a non-linear inverse relationship, with risk rising sharply below GNRI ≈ 100. Adding GNRI to a base clinical model significantly improved Harrell's C-index for MACE (Δ +0.048, *P* < 0.001) with excellent calibration.

**Conclusions:**

Lower GNRI is independently associated with adverse outcomes across the CKM spectrum. In an external CKM Stage 4 PCI cohort, GNRI provided clinically meaningful incremental risk discrimination, supporting its use as an inexpensive marker to help identify higher-risk patients. Whether GNRI-guided management improves outcomes remains to be determined.

## 1 Introduction

Metabolic abnormalities, chronic kidney disease (CKD), and cardiovascular disease (CVD) interact with each other closely, becoming a point of medical research (1, 2). The clustering of these conditions accelerates atherosclerosis, ventricular dysfunction, and progressive kidney dysfunction, resulting in bidirectional cardiovascular and renal injury, which contributes substantially to adverse health outcomes worldwide (3). To describe the shared pathophysiology and clinical overlap among metabolic disorders, CKD, and CVD, the American Heart Association formally introduced the construct of cardiovascular–kidney–metabolic (CKM) syndrome and proposed a four-stage framework that progresses from sub-clinical metabolic risk (Stage 1) to overt clinical CVD with concomitant kidney or metabolic disease (Stage 4) (4–6).

Population estimates indicate that nearly 90% of US adults meet criteria for CKM Stages 1–4, and prevalence continues to rise globally, underscoring the urgent need for early intervention and better risk stratification tools (7). Recent studies have evaluated biomarkers associated with prognosis in CKM syndrome. For instance, Zhang et al. examined the association between glucose associated indices and death due to CVD in a cohort with CKM syndrome, suggesting that these indices may offer valuable insights into the prognosis of CKM stages 0–3 (8). Additionally, Tang et al. explored the role of serum Klotho as a potential predictor of CKM severity and mortality, highlighting its association with both severity and adverse cardiovascular outcomes (9). Furthermore, Cao et al. studied inflammatory markers and CKM with mortality risk, emphasizing inflammatory factors in forecasting death due to CVD (10). Although these studies provide promising insights, there are notable limitations (11). For instance, the studies primarily focus on specific biomarkers without considering the broader spectrum of CKM syndrome-related risk factors. This limitation is particularly relevant in CKM syndrome, where patients range from sub-clinical metabolic disease to clinically established CVD, making it difficult for any single index to maintain consistent prognostic performance across the entire CKM continuum. There remains a particular lack of comprehensive risk prediction tools that integrate metabolic, renal, and cardiovascular clinical features in the highest-risk subgroup of CKM patients undergoing percutaneous coronary intervention (PCI).

The Geriatric Nutritional Risk Index (GNRI), originally derived from serum albumin and body weight, integrates nutritional status and subclinical inflammation into a single, easily computable score (12–14). This index, which reflects nutritional risk, has shown predictive value in various clinical settings, particularly in cardiovascular disease. Lower GNRI has been linked to adverse outcomes in heart failure (15, 16), post-PCI ischaemic heart failure (17), and CKD (13), with broader prognostic relevance suggested across other cardiovascular settings. Together, existing evidence suggests that GNRI may have potential value for risk assessment in cardiometabolic disease (18). However, no study to date has systematically evaluated the prognostic value of GNRI across the full CKM spectrum. Moreover, whether GNRI provides additional prognostic information beyond conventional clinical risk factors in high-risk CKM Stage 4 patients undergoing PCI remains unclear.

Accordingly, this study aimed to evaluate the prognostic value of GNRI across the CKM continuum using a two-phase discovery–validation design. In the discovery phase, we examined the association between GNRI and long-term mortality in a nationally representative population. In the validation phase, we assessed the incremental predictive performance of GNRI for adverse cardiovascular outcomes in a high-risk clinical cohort of CKM Stage 4 patients undergoing PCI. By combining population-based analysis with external validation in a high-risk PCI cohort, this study aimed to evaluate the consistency and clinical relevance of the GNRI–outcome association across different CKM populations.

## 2 Methods

### 2.1 Study design and ethics

This study comprised two complementary cohort analyses within a discovery–validation framework. The discovery analysis was a retrospective study of nationally representative survey data, while the external validation analysis was based on a contemporaneous, single-centre clinical cohort. Both phases were conducted in accordance with the Declaration of Helsinki. The discovery analysis used publicly available, de-identified NHANES data and was therefore exempt from institutional review board approval. The external validation study was approved by the Ethics Committee of Beijing Luhe Hospital, Capital Medical University, with waiver of informed consent owing to the retrospective use of de-identified electronic medical records.

### 2.2 Discovery cohort

#### 2.2.1 Data source

Discovery data were drawn from the National Health and Nutrition Examination Survey (NHANES), a continuous, multistage probability sample of the non-institutionalised civilian US population. NHANES collects detailed health and nutritional information through standardised questionnaires, physical examinations, and laboratory measurements (19). These data included demographic information, comorbidities, 10-year CVD risk score, vital signs, laboratory measurements.

#### 2.2.2 Study population

**Figure 1:**
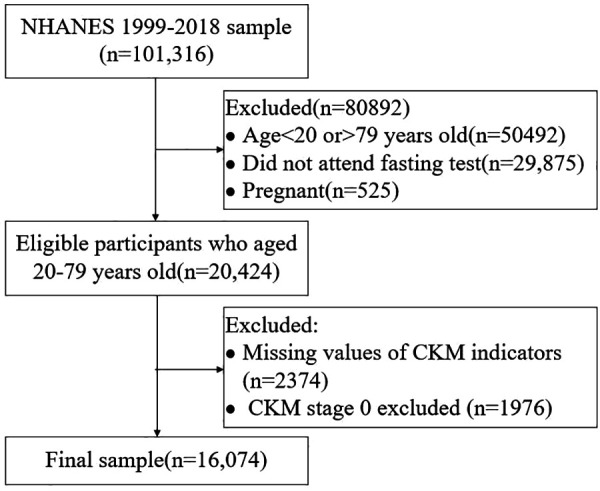
Flowchart of participant selection and exclusion criteria for the discovery cohort. CKM, cardiovascular–kidney–metabolic.

From the 1999–2018 cycles (*n* = 101,316), we sequentially excluded participants younger than 20 or older than 79 years (*n* = 50,492), those without a fasting laboratory specimen (*n* = 29,875), and pregnant women (*n* = 525), resulting in 20,424 eligible participants. We further excluded those with missing CKM-defining variables (*n* = 2,374) and those classified as CKM Stage 0 (*n* = 1,976), yielding a final analytic sample of 16,074 adults with CKM Stages 1–4 (Figure 1). CKM stages were assigned according to the American Heart Association definitions (2, 4, 6); sub-clinical CVD was indicated by a 10-year predicted CVD risk ≥20% (20) or by high-risk CKD.

#### 2.2.3 Exposure

GNRI was calculated using the formula of Bouillanne et al. (21): GNRI = [1.489 × serum albumin (g/L)] + [41.7 × (body weight/ideal body weight)], with the ratio of body weight to ideal body weight set to 1 when actual body weight exceeded ideal body weight. As is common in Asian cohorts, ideal body weight was estimated as 22 × [height (m)]^2^, corresponding to a body-mass index of 22 kg/m^2^ (22), rather than from the Lorenz equation used in the original description. Participants were grouped into NHANES-derived quartiles: Q1 (GNRI < 101.26), Q2 (101.26 ≤ GNRI < 104.24), Q3 (104.24 ≤ GNRI < 107.22), and Q4 (GNRI ≥ 107.22).

#### 2.2.4 Outcomes

The primary outcome was all-cause mortality. Secondary outcomes were cardiovascular and cancer mortality. Mortality was ascertained through linkage to the National Death Index (last update 31 December 2019), with cause of death classified using the International Classification of Diseases, 10th Revision (ICD-10) (23).

### 2.3 External validation cohort

#### 2.3.1 Setting and participants: CKM stage 4 patients undergoing PCI

The validation cohort consisted of consecutive adults who underwent PCI at Beijing Luhe Hospital. Because all participants had clinically manifest atherosclerotic cardiovascular disease (the indication for PCI) together with concomitant metabolic or renal abnormalities (47.4% diabetes mellitus, 22.1% hypertension at admission, 13.0% chronic kidney disease, and 23.5% obesity by Chinese criteria), the cohort corresponded to CKM Stage 4 within the AHA framework (2, 4). Inclusion required successful PCI, availability of admission serum albumin and anthropometric data needed to compute GNRI, and at least one follow-up assessment. Successful PCI was defined as angiographic success of the target lesion, with a residual diameter stenosis <30% and restoration of Thrombolysis in Myocardial Infarction (TIMI) grade 3 flow in the treated vessel, in the absence of major procedural complications. Among 2,979 patients who underwent successful PCI, we excluded those with a history of coronary artery bypass grafting (*n* = 79), missing data precluding GNRI calculation (*n* = 187), severe hepatic dysfunction (*n* = 57), or active malignancy with limited life expectancy <12 months (*n* = 13), as well as those lost to follow-up (*n* = 242), leaving 2,401 patients for analysis (Supplementary Figure S6).

#### 2.3.2 Definitions

Diabetes mellitus was defined as a documented diagnosis, ongoing glucose-lowering therapy, fasting plasma glucose ≥7.0 mmol/L, or glycated haemoglobin (HbA1c) ≥ 6.5%, in accordance with current American Diabetes Association criteria (24). Hypertension was defined as a documented diagnosis of hypertension, current use of antihypertensive medication, or an admission blood pressure ≥140/90 mmHg, in accordance with the diagnostic threshold of the 2024 ESC Guidelines for the management of elevated blood pressure and hypertension (25). CKD was defined as an estimated glomerular filtration rate (eGFR) < 60 mL/min/1.73 m^2^ calculated by the CKD-EPI equation. Obesity was defined as body mass index (BMI) ≥ 28 kg/m^2^ in accordance with Chinese criteria. Three implausible BMI values exceeding 50 kg/m^2^ were treated as data-entry errors and set to missing prior to analysis.

#### 2.3.3 Exposure

GNRI was calculated using serum albumin and body weight measured at admission, before the index PCI procedure, with the same formula as in the discovery cohort. Cohort-specific quartiles were used to preserve internal distributional balance: Q1 (GNRI ≤ 99.0), Q2 (99.1–104.0), Q3 (104.1–108.0), and Q4 (>108.0).

#### 2.3.4 Outcomes

The pre-specified primary outcome was a composite major adverse cardiovascular event (MACE) defined as the first occurrence of all-cause death, non-fatal myocardial infarction, or ischaemia-driven repeat revascularisation. Each component was also analysed separately as a secondary endpoint. Events were ascertained from in-hospital records and structured outpatient or telephone follow-up by trained nurses, and adjudicated by two independent cardiologists blinded to GNRI status, with disagreements resolved by consensus.

### 2.4 Statistical analysis

In the discovery cohort, continuous variables were summarised as median (interquartile range, IQR) and categorical variables as counts (weighted percentages); group comparisons across GNRI quartiles employed the Kruskal–Wallis test and the *χ*^2^ test, respectively. The relationship between GNRI and each outcome was assessed using Cox proportional hazards regression. Three sequential models were specified: Model 1 (unadjusted); Model 2 (adjusted for age, sex, and race); and Model 3 (fully adjusted for age, sex, race, education level, current smoking status, systolic blood pressure, waist circumference, low-density lipoprotein cholesterol [LDL-C], eGFR, and urine albumin-to-creatinine ratio [UACR]). Covariates for the fully adjusted model were specified *a priori* on the basis of established cardiovascular, kidney, and metabolic risk factors, biological plausibility, and the covariate sets used in previous NHANES-based CKM studies (5, 26, 27); established risk factors were retained regardless of their statistical significance, and their univariable associations with the outcome (*P* < 0.10) were examined in a supporting role. Restricted cubic spline (RCS) curves with four knots characterised the non-linear shape of the GNRI–outcome relationship. Subgroup analyses were performed across pre-defined strata of age, sex, race, education, smoking, clinical CVD, and 10-year CVD risk. All analyses in the discovery cohort incorporated NHANES sampling weights, strata, and primary sampling units in accordance with NHANES analytic guidelines.

In the external validation cohort, continuous variables were summarised as mean ± standard deviation or median [IQR] depending on distribution, and categorical variables as *n* (%); group differences across GNRI quartiles employed analysis of variance, Kruskal–Wallis, or *χ*^2^ tests as appropriate. Time to event was computed in years (1 month = 1/12 year), measured from the index PCI date. Event-free probability was estimated using the Kaplan–Meier method, with between-quartile comparisons by the log-rank test. Cox proportional hazards regression was again specified in three sequential models: Model 1 (unadjusted), Model 2 (adjusted for age and sex), and Model 3 (fully adjusted for age, sex, systolic blood pressure, BMI, LDL-C, eGFR, and glycated haemoglobin [HbA1c]). To keep the two cohorts comparable, the fully adjusted model in the validation cohort was harmonised, as far as the available data allowed, with the covariate set used in the discovery cohort, and was likewise specified *a priori* from established cardiovascular and post-PCI prognostic risk factors of recognised clinical relevance; the covariate set was adapted to the variables captured in this cohort. Established risk factors were retained irrespective of their statistical significance, with their univariable associations with the outcome (*P* < 0.10) examined in a supporting role. The proportional-hazards assumption was tested using scaled Schoenfeld residuals; no material violation was detected. RCS curves (four knots, reference at the median GNRI of 103.8) characterised dose–response relationships.

Incremental predictive performance conferred by GNRI in the validation cohort was evaluated using discrimination and calibration metrics. Harrell's concordance index (C-index) was estimated for a base clinical model including age, sex, systolic blood pressure, BMI, LDL-C, eGFR, and HbA1c, and then recalculated after adding GNRI as a continuous covariate. The absolute change in C-index (ΔC) and its 95% confidence interval were derived from 1,000 bootstrap replicates, and statistical significance was assessed using Somers' Dxy comparison. A ΔC ≥ 0.01 was considered statistically meaningful, whereas ΔC ≥ 0.02 was considered clinically meaningful. Time-dependent AUCs were calculated at 1, 2, and 3 years using the inverse probability of censoring weighting (IPCW) approach implemented in the timeROC package; the 3-year estimate was evaluated at 2.9 years to avoid numerical instability at the boundary of follow-up. Calibration was assessed by grouping patients into quartiles of model-predicted risk and comparing predicted risks with Kaplan–Meier–observed risks at 1, 2, and 3 years, with 95% confidence intervals derived from bootstrap resampling.

Subgroup analyses in the validation cohort evaluated the association between each 1-SD decrement in GNRI and MACE across pre-specified strata of age (<65 vs. ≥65 years), sex, glycaemic control (baseline HbA1c < 6.5% vs. ≥6.5%), hypertension, renal function (eGFR ≥ 60 vs. <60 mL/min/1.73 m^2^), and BMI (<28 vs. ≥28 kg/m^2^); interaction terms tested effect modification.

In Model 3 of the validation cohort, listwise deletion of participants with missing covariate data (*n* = 137; 5.7%) yielded a complete-case analytical sample of 2,264 patients; sensitivity analyses using multiple imputation produced concordant estimates. A two-sided *P* value <0.05 was considered statistically significant. Analyses were performed in Stata (MP 17.0; StataCorp, College Station, TX) and R 4.0.3 (R Foundation, Vienna, Austria). The R packages survival, rms, timeROC, pec, and ggplot2 were used for Cox regression, RCS modelling, time-dependent ROC analysis, calibration assessment, and visualisation, respectively.

## 3 Results

### 3.1 Part I—discovery cohort (NHANES, *n* = 16,074)

#### 3.1.1 Baseline characteristics

Of the 16,074 participants with CKM Stages 1–4, 52.2% (*n* = 8,338) were male and 47.8% (*n* = 7,736) female. Across ascending GNRI quartiles, the median of age, SBP, waist circumference, BMI, UACR, and HbA1c decreased significantly (all *P* < 0.001), as did the proportions of female participants, non-Hispanic Black participants, current smokers, and those classified as high or very-high CKD risk (Table 1). Conversely, education level, total and LDL-C, and serum creatinine increased across ascending GNRI quartiles. The prevalence of clinical comorbidities—including congestive heart failure, coronary heart disease, prior heart attack, and stroke—decreased monotonically across quartiles.

**Table 1:** Baseline characteristics of the discovery cohort (NHANES 1999–2018) stratified by GNRI quartiles.

Characteristic	Overall, *N* = 16,074	Quartiles of GNRI				*P* Value
		Q1	Q2	Q3	Q4	
		*N* = 4,894	*N* = 3,763	*N* = 3,710	*N* = 3,707	
Age, weighted median (IQR), y	48.00 (35.00, 59.00)	50.00 (38.00, 61.00)	50.00 (37.00, 61.00)	48.00 (36.00, 59.00)	43.00 (31.00, 55.00)	<0.001
Gender						<0.001
Male	8,338.0 (52.2%)	1,670.0 (31.9%)	1,752.0 (45.2%)	2,227.0 (59.8%)	2,689.0 (72.5%)	
Female	7,736.0 (47.8%)	3,224.0 (68.1%)	2,011.0 (54.8%)	1,483.0 (40.2%)	1,018.0 (27.5%)	
Race/Ethnicity						<0.001
Non-Hispanic White	6,736.0 (67.4%)	1,824.0 (62.7%)	1,547.0 (66.4%)	1,668.0 (70.6%)	1,697.0 (70.3%)	
Non-Hispanic Black	3,373.0 (11.5%)	1,451.0 (17.7%)	835.0 (12.3%)	604.0 (8.6%)	483.0 (6.9%)	
Mexican American	3,003.0 (8.5%)	778.0 (7.9%)	709.0 (8.4%)	736.0 (8.5%)	780.0 (9.4%)	
Other Race	1,521.0 (7.0%)	411.0 (6.3%)	325.0 (7.1%)	373.0 (6.9%)	412.0 (7.6%)	
Other Hispanic	1,441.0 (5.6%)	430.0 (5.5%)	347.0 (5.8%)	329.0 (5.3%)	335.0 (5.8%)	
Education Level, *n* (weighted %)						<0.001
<High school	4,345.0 (17.3%)	1,381.0 (18.8%)	1,030.0 (17.7%)	964.0 (16.3%)	970.0 (16.3%)	
High school	3,709.0 (24.5%)	1,162.0 (25.9%)	856.0 (23.9%)	851.0 (24.4%)	840.0 (23.8%)	
Some college or AA degree	4,558.0 (30.9%)	1,445.0 (32.1%)	1,055.0 (30.1%)	1,069.0 (31.6%)	989.0 (29.6%)	
College graduate or above	3,452.0 (27.2%)	901.0 (23.1%)	822.0 (28.3%)	824.0 (27.7%)	905.0 (30.2%)	
Current smoker, *n* (weighted %)	2,844.0 (17.5%)	987.0 (19.5%)	643.0 (17.2%)	600.0 (17.0%)	614.0 (16.3%)	0.028
CKM indicators						
Clinical CVD	1,552.0 (7.9%)	632.0 (11.2%)	373.0 (8.1%)	301.0 (7.0%)	246.0 (5.3%)	<0.001
Congestive heart failure, *n* (weighted %)	468.0 (2.2%)	225.0 (3.7%)	106.0 (2.2%)	72.0 (1.4%)	65.0 (1.3%)	<0.001
Coronary heart disease, *n* (weighted %)	636.0 (3.5%)	220.0 (4.4%)	151.0 (3.4%)	146.0 (3.5%)	119.0 (2.5%)	0.004
Heart attack, *n* (weighted %)	701.0 (3.6%)	275.0 (4.7%)	176.0 (4.0%)	136.0 (3.2%)	114.0 (2.4%)	<0.001
Stroke, *n* (weighted %)	535.0 (2.6%)	245.0 (4.3%)	128.0 (2.6%)	94.0 (2.0%)	68.0 (1.5%)	<0.001
CKD risk, *n* (weighted %)						
Moderate	1,870.0 (9.5%)	697.0 (12.0%)	444.0 (10.0%)	382.0 (8.7%)	347.0 (7.4%)	<0.001
High	409.0 (1.8%)	220.0 (3.8%)	79.0 (1.3%)	68.0 (1.1%)	42.0 (0.7%)	<0.001
Very high	196.0 (0.7%)	134.0 (1.6%)	27.0 (0.5%)	22.0 (0.3%)	13.0 (0.1%)	<0.001
Metabolic disorders, *n*, (weighted %)						
Overweight/obesity	12,756.0 (78.7%)	4,055.0 (82.6%)	3,066.0 (80.0%)	2,916.0 (78.7%)	2,719.0 (73.4%)	<0.001
Abdominal obesity	10,201.0 (62.8%)	3,679.0 (75.8%)	2,542.0 (66.9%)	2,220.0 (60.3%)	1,760.0 (47.8%)	<0.001
Prediabetes	7,640.0 (47.8%)	2,178.0 (45.7%)	1,784.0 (47.8%)	1,878.0 (50.6%)	1,800.0 (47.5%)	0.014
Diabetes	2,940.0 (13.8%)	1,193.0 (19.4%)	680.0 (13.5%)	585.0 (12.0%)	482.0 (9.7%)	<0.001
Hypertriglyceridemia	6,022.0 (37.9%)	1,696.0 (36.7%)	1,344.0 (34.9%)	1,457.0 (39.5%)	1,525.0 (40.2%)	<0.001
Hypertension	9,528.0 (55.8%)	3,110.0 (60.2%)	2,218.0 (55.2%)	2,147.0 (55.2%)	2,053.0 (52.5%)	<0.001
MetS	7,206.0 (42.6%)	2,507.0 (49.9%)	1,703.0 (42.8%)	1,633.0 (42.8%)	1,363.0 (34.6%)	<0.001
10-y CVD risk score, weighted median (IQR), %	2.71 (0.83, 7.78)	3.45 (1.02, 9.97)	3.24 (0.96, 8.41)	2.70 (0.89, 7.32)	1.86 (0.65, 5.31)	<0.001
CKM stage						<0.001
Stage 1	3,591.0 (24.8%)	910.0 (20.4%)	875.0 (26.1%)	884.0 (26.4%)	922.0 (26.7%)	
Stage 2	10,041.0 (63.9%)	3,004.0 (63.9%)	2,292.0 (62.2%)	2,335.0 (63.6%)	2,410.0 (65.8%)	
Stage 3	890.0 (3.4%)	348.0 (4.5%)	223.0 (3.6%)	190.0 (3.0%)	129.0 (2.3%)	
Stage 4	1,552.0 (7.9%)	632.0 (11.2%)	373.0 (8.1%)	301.0 (7.0%)	246.0 (5.3%)	
Vital signs						
SBP, weighted median (IQR), mmHg	121.33 (112.00, 132.00)	122.00 (112.00, 134.00)	120.67 (111.33, 131.33)	120.67 (112.00, 131.33)	121.33 (112.67, 131.33)	0.015
DBP, weighted median (IQR), mmHg	72.00 (65.33, 79.33)	72.00 (64.67, 79.33)	72.00 (64.67, 78.00)	72.00 (64.67, 78.67)	72.67 (66.00, 79.33)	0.011
Waist circumference, weighted median (IQR), cm	99.50 (90.50, 110.00)	104.20 (93.30, 116.80)	99.90 (91.00, 110.50)	99.00 (90.40, 108.50)	96.70 (88.80, 105.20)	<0.001
BMI, weighted median (IQR), kg/m^2^	28.64 (25.40, 33.00)	31.20 (26.45, 36.67)	28.70 (25.52, 33.40)	28.28 (25.30, 32.00)	27.30 (24.66, 30.39)	<0.001
Laboratory measurements						
TG, weighted median (IQR), mg/dL	112.00 (77.00, 163.00)	110.00 (76.00, 158.00)	108.00 (75.00, 158.00)	115.00 (78.00, 166.00)	117.00 (79.00, 171.00)	<0.001
TC, weighted median (IQR), mg/dL	195.00 (170.00, 222.00)	190.00 (165.00, 216.00)	194.00 (170.00, 220.00)	196.00 (171.00, 223.00)	199.00 (173.00, 228.00)	<0.001
LDL-C, weighted median (IQR), mg/dL	116.00 (94.00, 140.00)	111.00 (90.00, 134.00)	115.00 (94.00, 138.00)	117.00 (95.00, 141.00)	121.00 (98.00, 146.00)	<0.001
HDL-C, weighted median (IQR), mg/dL	51.00 (42.00, 61.00)	51.00 (42.00, 62.00)	51.00 (43.00, 62.00)	50.00 (42.00, 60.00)	50.00 (42.00, 60.00)	<0.001
Creatinine, weighted median (IQR), mg/dL	0.84 (0.70, 1.00)	0.80 (0.70, 0.93)	0.82 (0.70, 0.99)	0.88 (0.73, 1.00)	0.90 (0.77, 1.00)	<0.001
eGFR, weighted median (IQR), mL/min × 1.73 m^2^	99.05 (85.10, 111.42)	97.90 (82.68, 109.98)	97.63 (83.78, 109.98)	98.72 (85.02, 110.73)	102.51 (89.36, 114.63)	<0.001
Urinary albumin/creatinine ratio, weighted median (IQR), mg/g	6.17 (4.12, 11.22)	6.80 (4.46, 13.80)	6.34 (4.26, 10.96)	5.85 (4.00, 10.59)	5.63 (3.95, 10.07)	<0.001
FBG, weighted median (IQR), mg/dL	100.00 (93.30, 109.00)	101.00 (93.00, 111.30)	100.00 (93.00, 109.00)	101.00 (94.00, 109.00)	100.00 (93.80, 107.00)	0.001
HbA1c weighted median (IQR), %	5.40 (5.20, 5.70)	5.50 (5.30, 5.90)	5.40 (5.20, 5.80)	5.40 (5.20, 5.70)	5.30 (5.10, 5.60)	<0.001

Continuous variables are presented as median (interquartile range) and compared using the Kruskal–Wallis test. Categorical variables are presented as *n* (weighted %) and compared using the *χ*^2^ test.

BMI, body mass index; CKD, chronic kidney disease; CKM, cardiovascular–kidney–metabolic; CVD, cardiovascular disease; DBP, diastolic blood pressure; eGFR, estimated glomerular filtration rate; FBG, fasting blood glucose; GNRI, Geriatric Nutritional Risk Index; HbA1c, glycated haemoglobin; HDL-C, high-density lipoprotein cholesterol; IQR, interquartile range; LDL-C, low-density lipoprotein cholesterol; SBP, systolic blood pressure; UACR, urine albumin-to-creatinine ratio.

#### 3.1.2 Outcomes

All-cause mortality was inversely related to GNRI quartile (Q1, 11.3%; Q2, 10.5%; Q3, 8.7%; Q4, 6.9%; *P* < 0.001), with a similar pattern for cardiovascular mortality (3.3% to 1.5%; *P* < 0.001). Cancer mortality showed a non-significant trend toward higher rates in lower GNRI quartiles (*P* = 0.067; Supplementary Table S1).

#### 3.1.3 Cox regression

In Cox proportional hazards models, lower GNRI quartiles were generally associated with higher risks of all-cause and cardiovascular mortality across increasingly adjusted models, whereas the association with cancer mortality was attenuated after full adjustment (Supplementary Table S2). For all-cause mortality, the fully adjusted hazard ratio (Model 3) for Q1 vs. Q4 was 1.58 (95% CI 1.32–1.89) and for Q2 vs. Q4 was 1.31 (95% CI 1.09–1.57); the corresponding HR per 1-unit increase in GNRI was 0.95 (95% CI 0.94–0.97). For cardiovascular mortality, the fully adjusted Q1-vs.-Q4 HR was 2.13 (95% CI 1.55–2.93). For cancer mortality, the unadjusted Q1-vs.-Q4 association (HR 1.87; 95% CI 1.31–2.68) attenuated to non-significance after full adjustment (HR 1.41; 95% CI 0.98–2.02), although the continuous association remained statistically significant (HR per 1-unit GNRI 0.96; 95% CI 0.94–0.99).

#### 3.1.4 Non-linearity

RCS analyses confirmed an inverse, non-linear dose–response relationship between GNRI and all-cause mortality (*P*-non-linear < 0.0001), with hazard rising steeply below GNRI ≈ 100 (Supplementary Figure S1A). The associations with cardiovascular and cancer mortality were monotonic but did not reach formal statistical evidence of non-linearity (*P*-non-linear = 0.284 and 0.241, respectively; Supplementary Figures S1B,C).

#### 3.1.5 Subgroup analyses

The protective association of higher GNRI with all-cause mortality was robust across a wide range of subgroups, including demographic characteristics and clinical risk profiles (Supplementary Figure S2). Significant interactions were detected for sex (*P*-interaction < 0.001) and 10-year CVD risk score (*P*-interaction < 0.001). For cause-specific mortality (Supplementary Figure S3), GNRI remained inversely associated with cardiovascular and cancer mortality in most subgroups, with significant sex and 10-year CVD risk interactions for both endpoints. The predictive performance for cancer mortality was attenuated in women, non-Hispanic Whites, and participants with 10-year CVD risk ≥20%, suggesting that the association may vary across specific population subgroups.

### 3.2 Part II—external validation in CKM stage 4 patients undergoing PCI (*n* = 2,401)

#### 3.2.1 Baseline characteristics

The validation cohort comprised 2,401 patients (mean age 61.0 ± 11.0 years, 81.0% male) representing the most clinically advanced segment of the CKM continuum. Ascending GNRI quartiles were associated with younger age, higher BMI, higher serum albumin and haemoglobin, higher eGFR, and a higher proportion of male sex (all *P* < 0.001), whereas the prevalence of CKD decreased across quartiles (18.8% in Q1 vs. 9.7% in Q4; *P* < 0.001) (Table 2). Diabetes mellitus prevalence (47.4% overall) and LDL cholesterol did not differ materially across quartiles, indicating that GNRI primarily reflects nutritional status rather than glycaemic or lipid profiles.

**Table 2:** Baseline characteristics of the external validation cohort (CKM Stage 4, post-PCI) stratified by GNRI quartiles.

Variable	Overall, *N* = 2,401	Quartiles of GNRI				*P* value
		Q1	Q2	Q3	Q4	
		*N* = 611	*N* = 598	*N* = 602	*N* = 590	
Age, y	61.0 ± 11.0	65.5 ± 10.3	61.6 ± 10.1	60.3 ± 10.3	56.5 ± 11.4	<0.001
Male, *n* (%)	1,945 (81.0)	471 (77.1)	468 (78.3)	491 (81.6)	515 (87.3)	<0.001
BMI, kg/m^2^	25.8 ± 3.5	24.8 ± 3.8	25.7 ± 3.5	26.1 ± 3.4	26.5 ± 3.3	<0.001
SBP, mmHg	124.3 ± 18.4	121.3 ± 18.4	124.3 ± 17.4	124.7 ± 18.1	127.0 ± 19.1	<0.001
DBP, mmHg	74.0 ± 12.0	70.9 ± 11.3	74.0 ± 11.6	74.4 ± 12.1	76.6 ± 12.2	<0.001
Serum albumin, g/L	41.7 ± 4.0	36.7 ± 2.5	40.6 ± 1.0	43.0 ± 0.9	46.7 ± 1.9	<0.001
Haemoglobin, g/L	138.0 ± 18.7	128.8 ± 19.6	136.9 ± 18.0	140.9 ± 16.1	146.3 ± 16.0	<0.001
LDL-C, mmol/L	2.35 ± 0.87	2.30 ± 0.83	2.37 ± 0.89	2.39 ± 0.86	2.34 ± 0.91	0.380
GNRI	103.4 ± 6.3	95.3 ± 4.1	101.9 ± 1.2	105.5 ± 1.1	111.1 ± 2.8	<0.001
Serum creatinine, μmol/L, median (IQR)	77.0 (66.7–91.1)	78.5 (65.8–99.8)	76.3 (65.3–90.7)	76.0 (67.3–88.8)	77.0 (67.6–89.2)	0.143
eGFR, mL/min/1.73 m^2^, median (IQR)	90.5 (72.9–101.1)	86.8 (65.0–97.7)	90.3 (73.6–101.0)	91.6 (75.3–101.0)	93.3 (78.3–104.6)	<0.001
Diabetes mellitus, *n* (%)	1,139 (47.4)	307 (50.2)	278 (46.5)	290 (48.2)	264 (44.7)	0.260
Hypertension, *n* (%)	531 (22.1)	113 (18.5)	133 (22.2)	131 (21.8)	154 (26.1)	0.017
CKD, *n* (%)	313 (13.0)	115 (18.8)	69 (11.5)	72 (12.0)	57 (9.7)	<0.001
Obesity, *n* (%)	565 (23.5)	122 (20.0)	140 (23.4)	141 (23.4)	162 (27.5)	0.025

Continuous variables are presented as mean ± standard deviation and compared across GNRI quartiles using one-way analysis of variance, except for serum creatinine and eGFR, which are presented as median (interquartile range) and compared using the Kruskal–Wallis test owing to skewed distributions. Categorical variables are presented as *n* (%) and compared using the *χ*^2^ test.

BMI, body mass index; SBP, systolic blood pressure; DBP, diastolic blood pressure; eGFR, estimated glomerular filtration rate; GNRI, Geriatric Nutritional Risk Index; LDL-C, low-density lipoprotein cholesterol; CKD, chronic kidney disease.

#### 3.2.2 Outcomes

**Figure 2:**
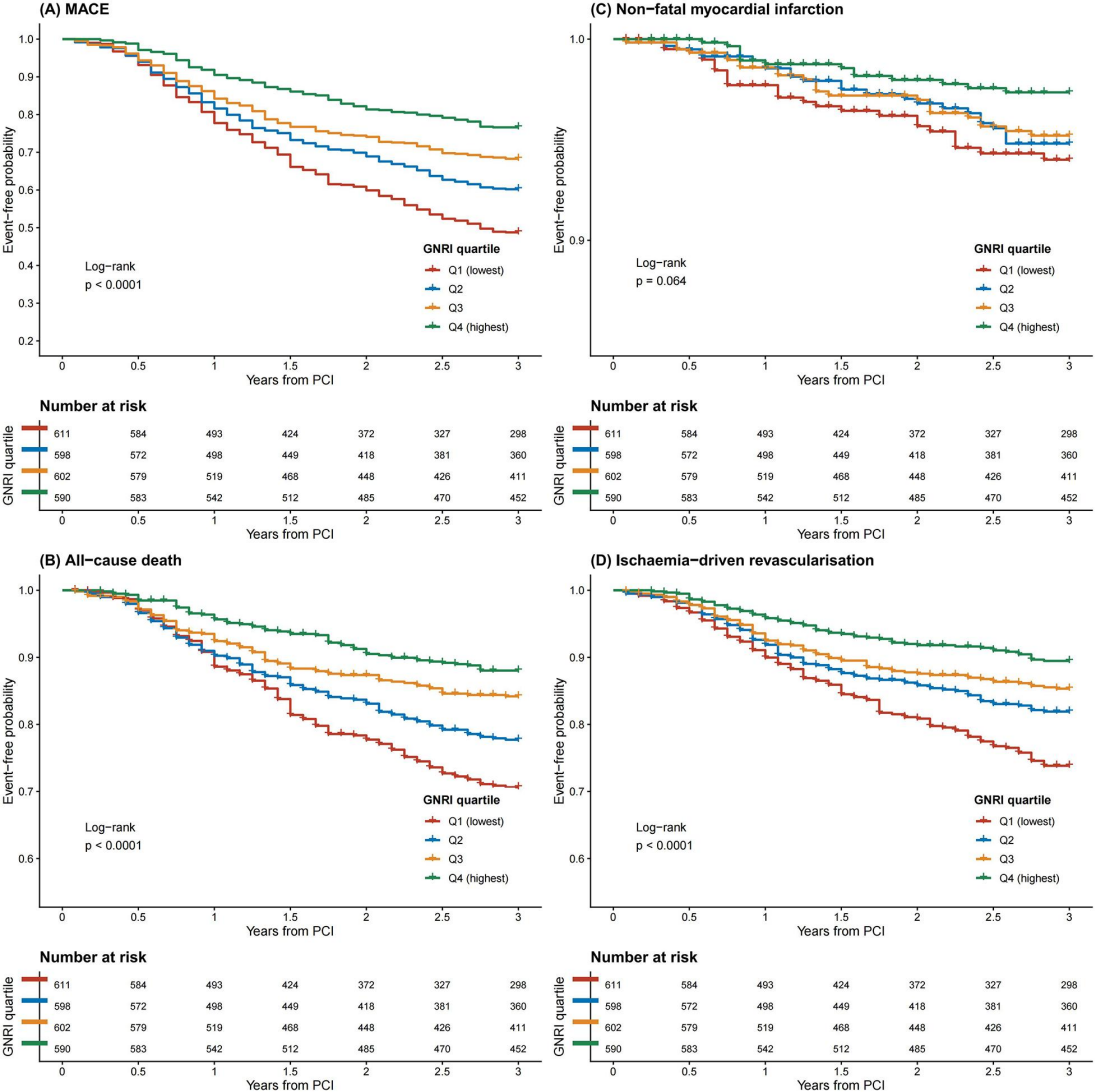
Kaplan–Meier event-free probability curves stratified by GNRI quartile in the external validation cohort of CKM stage 4 patients undergoing PCI: **(A)** MACE, **(B)** all-cause death, **(C)** non-fatal myocardial infarction, and **(D)** ischaemia-driven repeat revascularisation. Numbers at risk are shown beneath each panel. Log-rank *P* values: **(A)** <0.0001, **(B)** <0.0001, **(C)** 0.064, **(D)** <0.0001. CKM, cardiovascular–kidney–metabolic; GNRI, Geriatric Nutritional Risk Index; MACE, major adverse cardiovascular event; PCI, percutaneous coronary intervention; Q, quartile.

Over a median follow-up of 1.58 years (IQR 0.69–3.00) and 5,711.8 person-years of observation, 880 MACE events (36.7%), 424 all-cause deaths (17.7%), 90 non-fatal myocardial infarctions (3.7%), and 366 ischaemia-driven repeat revascularisations (15.2%) were recorded (Supplementary Table S3). Event rates increased monotonically across descending GNRI quartiles. The MACE incidence rate per 100 person-years was 23.88 (95% CI 21.38–26.68) in Q1 vs. 8.90 (95% CI 7.53–10.52) in Q4; cumulative MACE incidence at 3 years was 51.2% in Q1 vs. 23.4% in Q4. Kaplan–Meier curves demonstrated highly significant separation across GNRI quartiles for MACE, all-cause death, and ischaemia-driven revascularisation (all log-rank *P* < 0.001), and a borderline trend for non-fatal myocardial infarction (*P* = 0.064) (Figures 2A–D).

#### 3.2.3 Cox regression

In fully adjusted Cox models (Model 3, *n* = 2,264), patients in Q1 had a 2.52-fold higher hazard of MACE (95% CI 2.03–3.13), a 2.45-fold higher hazard of all-cause death (95% CI 1.80–3.34), a 2.03-fold higher hazard of non-fatal myocardial infarction (95% CI 1.03–4.02), and a 2.74-fold higher hazard of ischaemia-driven revascularisation (95% CI 1.96–3.83) compared with Q4 (Table 3). All *P*-for-trend values were <0.001 except for non-fatal myocardial infarction (*P*-trend = 0.037). The fully adjusted hazard ratio per 1-unit GNRI increase was 0.95 (95% CI 0.94–0.96) for MACE and directionally consistent and of similar magnitude for all-cause death and ischaemia-driven revascularisation, indicating a roughly 5% reduction in hazard for every 1-point gain in nutritional reserve.

**Table 3:** Hazard ratios for cardiovascular outcomes across GNRI quartiles.

GNRI category	Model 1 (Unadjusted) *n* = 2,401	Model 2 (Age, sex) *n* = 2,401	Model 3 (Fully adjusted) *n* = 2,264
MACE (events: 880/2,401 overall; 825/2,264 in Model 3)			
Q1 (lowest) vs. Q4	2.64 (2.16–3.22)	2.60 (2.11–3.20)	2.52 (2.03–3.13)
Q2 vs. Q4	1.92 (1.55–2.36)	1.92 (1.55–2.37)	1.91 (1.54–2.38)
Q3 vs. Q4	1.46 (1.18–1.82)	1.46 (1.17–1.82)	1.41 (1.13–1.77)
Q4 (highest, reference)	1.00 (Ref)	1.00 (Ref)	1.00 (Ref)
*P* for trend	<0.001	<0.001	<0.001
GNRI per 1-unit increase	0.95 (0.94–0.96)	0.95 (0.94–0.96)	0.95 (0.94–0.96)
All-cause death (events: 424/2,401 overall; 393/2,264 in Model 3)			
Q1 (lowest) vs. Q4	2.68 (2.01–3.58)	2.59 (1.92–3.49)	2.45 (1.80–3.34)
Q2 vs. Q4	2.00 (1.48–2.71)	1.98 (1.46–2.68)	1.87 (1.37–2.56)
Q3 vs. Q4	1.39 (1.01–1.92)	1.38 (1.00–1.90)	1.31 (0.95–1.82)
Q4 (highest, reference)	1.00 (Ref)	1.00 (Ref)	1.00 (Ref)
*P* for trend	<0.001	<0.001	<0.001
GNRI per 1-unit increase	0.95 (0.94–0.97)	0.95 (0.94–0.97)	0.95 (0.94–0.97)
Non-fatal myocardial infarction (events: 90/2,401 overall; 86/2,264 in Model 3)			
Q1 (lowest) vs. Q4	2.36 (1.24–4.48)	2.26 (1.16–4.39)	2.03 (1.03–4.02)
Q2 vs. Q4	1.92 (0.99–3.70)	1.91 (0.98–3.72)	1.95 (1.00–3.81)
Q3 vs. Q4	1.82 (0.94–3.51)	1.79 (0.93–3.48)	1.61 (0.82–3.17)
Q4 (highest, reference)	1.00 (Ref)	1.00 (Ref)	1.00 (Ref)
*P* for trend	0.020	0.025	0.037
GNRI per 1-unit increase	0.96 (0.93–0.99)	0.96 (0.93–0.99)	0.97 (0.93–1.00)
Ischaemia-driven repeat revascularisation (events: 366/2,401 overall; 346/2,264 in Model 3)			
Q1 (lowest) vs. Q4	2.66 (1.95–3.62)	2.70 (1.96–3.73)	2.74 (1.96–3.83)
Q2 vs. Q4	1.82 (1.31–2.52)	1.85 (1.33–2.57)	1.95 (1.39–2.73)
Q3 vs. Q4	1.46 (1.04–2.04)	1.47 (1.05–2.06)	1.48 (1.05–2.10)
Q4 (highest, reference)	1.00 (Ref)	1.00 (Ref)	1.00 (Ref)
*P* for trend	<0.001	<0.001	<0.001
GNRI per 1-unit increase	0.95 (0.94–0.97)	0.95 (0.94–0.97)	0.95 (0.94–0.97)

Data are presented as HR (95% CI) from Cox proportional hazards regression. Time was scaled to years (1 month = 1/12 year). Model 1: unadjusted. Model 2: adjusted for age and sex. Model 3: adjusted for age, sex, systolic blood pressure, BMI, LDL-C, eGFR, and HbA1c. The reduced sample size in Model 3 reflects listwise exclusion of participants with missing covariate data (*n* = 137; 5.7%). GNRI quartiles are defined within the analytical cohort: Q1 (lowest, poorest nutritional status) to Q4 (highest, best nutritional status; reference category). MACE was defined as a composite of all-cause death, non-fatal myocardial infarction, and ischaemia-driven repeat revascularisation. *P* for trend was estimated by modelling the GNRI quartile as an ordinal continuous variable in the corresponding Cox model. GNRI per 1-unit increase represents the hazard ratio for each 1-point increment in the continuous GNRI score; HRs < 1 indicate that higher GNRI (better nutrition) is associated with lower event risk.

BMI, body mass index; CI, confidence interval; CKM, cardiovascular–kidney–metabolic; eGFR, estimated glomerular filtration rate; GNRI, Geriatric Nutritional Risk Index; HbA1c, glycated haemoglobin; HR, hazard ratio; LDL-C, low-density lipoprotein cholesterol; MACE, major adverse cardiovascular event; PCI, percutaneous coronary intervention; Q, quartile; SBP, systolic blood pressure.

#### 3.2.4 Non-linear dose–response

**Figure 3:**
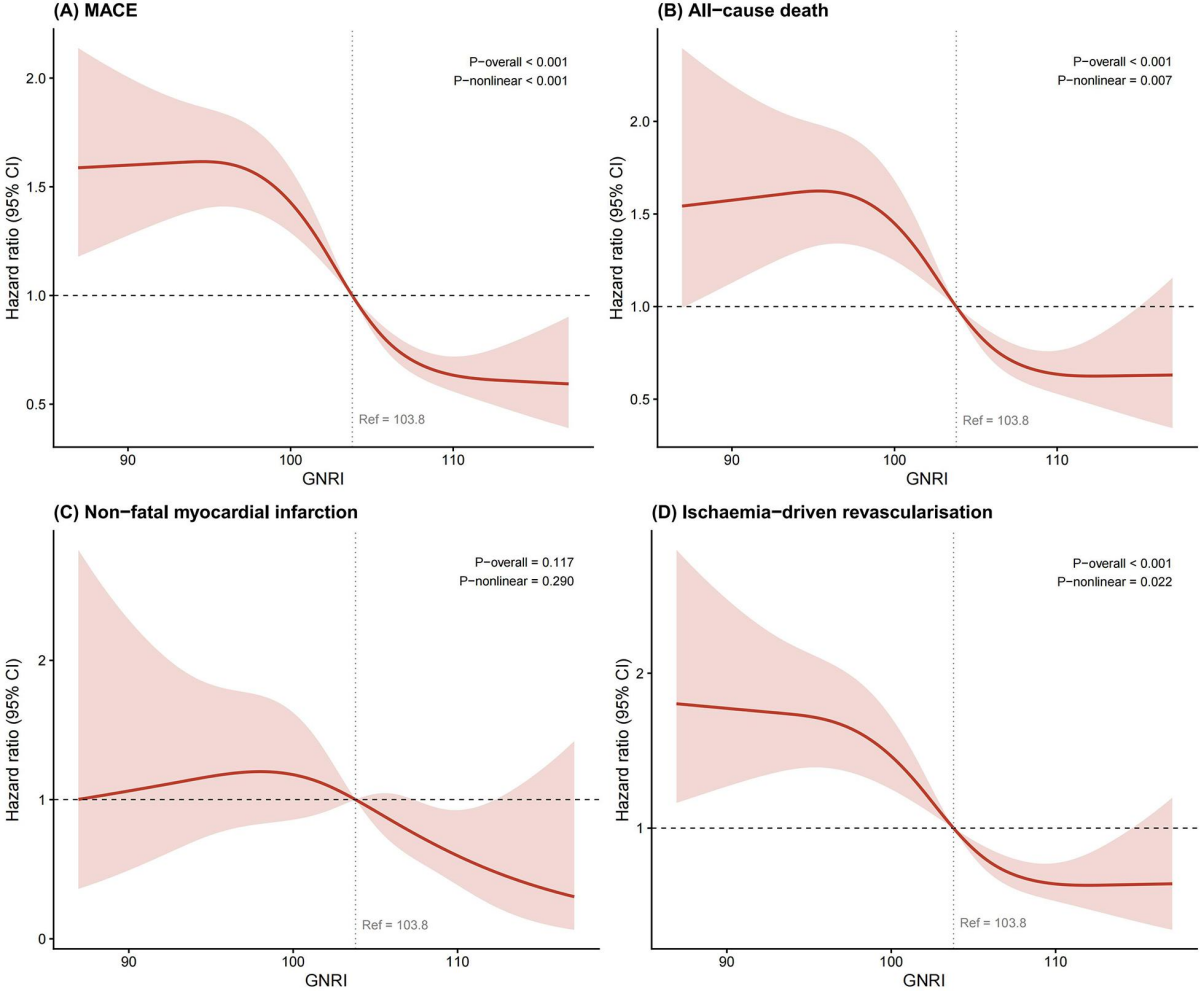
Restricted cubic spline curves modelling the dose–response relationship between GNRI and cardiovascular outcomes in the external validation cohort: **(A)** MACE, **(B)** all-cause death, **(C)** non-fatal myocardial infarction, and **(D)** ischaemia-driven repeat revascularisation. The reference value was set at the cohort median GNRI of 103.8. The shaded area represents the 95% confidence interval. Overall and/or non-linear *P* values are shown for each panel where applicable. CI, confidence interval; GNRI, Geriatric Nutritional Risk Index; HR, hazard ratio; MACE, major adverse cardiovascular event.

RCS analyses (reference GNRI = 103.8) confirmed a strongly non-linear inverse association between GNRI and MACE (*P*-overall < 0.001, *P*-non-linear < 0.001), with hazard rising sharply below GNRI ≈ 100 (Figure 3A). Comparable non-linear patterns were observed for all-cause death (*P*-non-linear = 0.007; Figure 3B) and ischaemia-driven revascularisation (*P*-non-linear = 0.022; Figure 3D). For non-fatal myocardial infarction, the association was directionally consistent but did not achieve statistical significance (*P*-overall = 0.117; Figure 3C), in keeping with the smaller event count.

#### 3.2.5 Discrimination and incremental value

Adding GNRI to a base clinical model substantially improved discrimination for MACE (Harrell's C-index 0.566 → 0.614; ΔC +0.048, 95% CI +0.029 to +0.067; *P* < 0.001), all-cause death (0.570 → 0.612; ΔC +0.042; *P* = 0.003), and ischaemia-driven revascularisation (0.562 → 0.612; ΔC +0.050; *P* < 0.001) (Table 4). The improvement for non-fatal myocardial infarction was directionally consistent but did not achieve statistical significance (ΔC +0.023; *P* = 0.426), again likely reflecting the limited number of events. Time-dependent ROC analysis demonstrated that the base + GNRI model achieved AUCs of 0.616, 0.630, and 0.645 at 1, 2, and 3 years, respectively, with the GNRI-augmented model uniformly outperforming the base model across the entire follow-up window (Supplementary Figures S4A,B). All ΔC estimates for MACE, all-cause death, and ischaemia-driven revascularisation exceeded the 0.02 threshold for clinical meaningfulness in cardiovascular prediction modelling.

**Table 4:** Incremental predictive value of GNRI for cardiovascular outcomes.

Panel A. Harrell's C-index (overall discrimination across follow-up)					
Outcome	Events/*N*	Base model C-index (95% CI)	Base + GNRI C-index (95% CI)	ΔC-index (95% CI)	*P* value
MACE	825/2,264	0.566 (0.546–0.586)	0.614 (0.595–0.633)	+0.048 (+0.029 to +0.067)	<0.001
All-cause death	393/2,264	0.570 (0.540–0.600)	0.612 (0.584–0.640)	+0.042 (+0.014 to +0.070)	0.003
Non-fatal myocardial infarction	86/2,264	0.604 (0.545–0.664)	0.627 (0.569–0.685)	+0.023 (−0.034 to +0.079)	0.426
Ischaemia-driven repeat revascularisation	346/2,264	0.562 (0.530–0.594)	0.612 (0.583–0.642)	+0.050 (+0.021 to +0.080)	<0.001
Panel B. Time-dependent AUC for MACE (primary outcome)					
Time horizon			AUC (Base + GNRI)		
1-year			0.616		
2-year			0.630		
3-year (evaluated at 2.9 years)			0.645		

All analyses were performed in the complete-case cohort (*n* = 2,264) with non-missing values for all covariates included in the multivariable models. Base model: Cox proportional hazards model adjusted for age, sex, systolic blood pressure, body mass index, low-density lipoprotein cholesterol, estimated glomerular filtration rate, and glycated haemoglobin. Base + GNRI model: base model with GNRI added as a continuous covariate. Harrell's C-index summarises overall discrimination across the entire follow-up. ΔC-index represents the absolute increase in C-index after adding GNRI; the corresponding *P* value tests the null hypothesis of no improvement (Somers' Dxy difference test). Time-dependent AUC was computed using the inverse probability of censoring weighting (IPCW) approach (timeROC package); the 3-year estimate was evaluated at 2.9 years to avoid numerical instability at the boundary of follow-up. Reference thresholds for the magnitude of ΔC-index in cardiovascular prediction modelling: ΔC ≥ 0.01 is considered statistically meaningful, and ΔC ≥ 0.02 is considered clinically meaningful.

AUC, area under the curve; CI, confidence interval; CKM, cardiovascular–kidney–metabolic; GNRI, Geriatric Nutritional Risk Index; MACE, major adverse cardiovascular event; PCI, percutaneous coronary intervention; Δ, change.

#### 3.2.6 Model calibration

**Figure 4:**
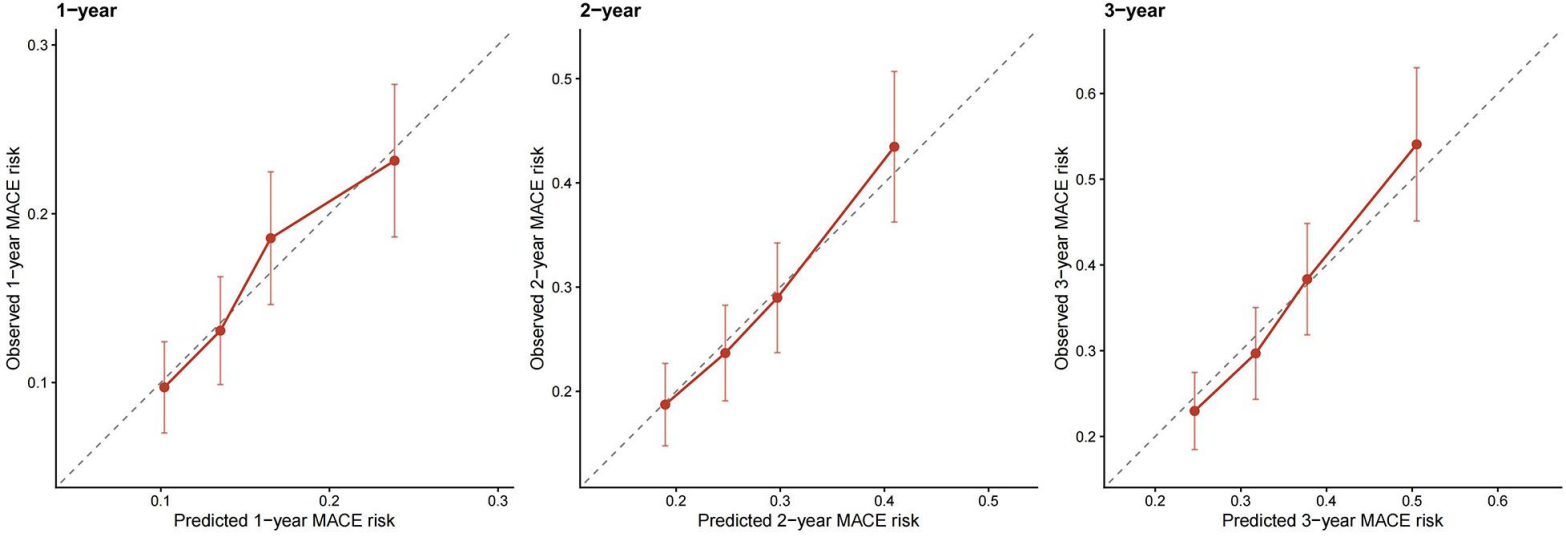
Calibration plots of the base + GNRI Cox model for MACE in the external validation cohort at 1, 2, and 3 years. Each point represents a quartile of predicted risk; the dashed diagonal denotes perfect calibration. Observed risks (estimated by the Kaplan–Meier method, with 95% confidence intervals from 1,000 bootstrap replications) closely approximated predicted risks across all quartiles and time horizons, with absolute deviations below 5%. GNRI, Geriatric Nutritional Risk Index; MACE, major adverse cardiovascular event.

Calibration of the base + GNRI Cox model was good across all three time horizons (Figure 4; numerical values are provided in Supplementary Table S4). Predicted and observed MACE risks aligned closely across quartiles of predicted risk: at 1 year, absolute differences ranged from −0.7% to +2.0%; at 2 years, from −1.0% to +2.5%; and at 3 years, from −2.1% to +3.5%. All twelve quartile × time-point combinations had absolute deviations below the 5% threshold, and 95% confidence intervals of the observed estimates encompassed the predicted values, supporting accurate risk estimation in this external cohort.

#### 3.2.7 Subgroup analysis

In subgroup analyses, 2,263 patients had complete data for all stratifying variables; one additional participant was excluded because of missing data for at least one stratifying variable, resulting in a sample size one smaller than that used for the complete-case Model 3 analysis. Each 1-SD decrement in GNRI conferred an HR of 1.35 (95% CI 1.26–1.44) for MACE in the overall cohort (Supplementary Figure S5). The association was directionally consistent across all six pre-specified strata, including age (HR 1.46 in <65 years vs. 1.32 in ≥65 years; *P*-interaction = 0.509), sex (1.33 in men vs. 1.38 in women; *P*-interaction = 0.386), hypertension status (1.36 in non-hypertensive vs. 1.36 in hypertensive patients; *P*-interaction = 0.628), renal function (1.35 vs. 1.42 in eGFR ≥60 vs. <60 mL/min/1.73 m^2^; *P*-interaction = 0.734), and BMI (1.37 vs. 1.27 in BMI <28 vs. ≥28 kg/m^2^; *P*-interaction = 0.117). A statistically significant interaction was detected for glycaemic control (HR 1.27 for HbA1c <6.5% vs. HR 1.47 for HbA1c ≥6.5%; *P*-interaction = 0.012), indicating that the prognostic value of GNRI was more pronounced in patients with poor glycaemic control, although the protective direction was preserved in both strata.

## 4 Discussion

This two-cohort discovery–validation study demonstrated that lower GNRI was independently associated with all-cause and cardiovascular mortality across the full CKM continuum in a nationally representative sample of nearly 16,000 US adults. In an external validation cohort of 2,401 Chinese patients with CKM Stage 4 undergoing PCI, the same exposure was dose-dependently associated with MACE, all-cause death, and ischaemia-driven revascularisation. Adding GNRI to a comprehensive clinical risk model produced a clinically meaningful improvement in discrimination together with good calibration across follow-up. Together, the convergence of population-level and clinical-cohort evidence supports GNRI as a readily calculable and potentially actionable prognostic tool within the CKM framework, with particular utility in patients at greatest absolute risk.

GNRI was originally developed to overcome the limitations of using BMI or serum albumin in isolation, integrating nutritional reserve and a low-grade systemic inflammatory state into a single, easily computed score (21). Its prognostic relevance in cardiovascular disease is well established: malnutrition amplifies cardiovascular risk through systemic inflammation, accelerated atherosclerosis, and impaired myocardial energetics (28–30). In the validation cohort, a low GNRI was associated not only with mortality but also with non-fatal myocardial infarction and ischaemia-driven revascularisation, and several mechanisms may account for these ischaemic endpoints. Inflammation and oxidative stress promote endothelial dysfunction, plaque instability, and a prothrombotic state that favours recurrent atherothrombotic events. Hypoalbuminaemia is associated with high on-treatment platelet reactivity and a weaker antiplatelet response to aspirin (31), and poor nutritional status independently predicts repeat revascularisation and restenosis after PCI (32). The body-weight component of GNRI may further reflect reduced muscle mass, sarcopenia, and frailty, which are themselves linked to adverse cardiovascular outcomes and multi-organ decline (18, 33–35). However, because neither cohort included a direct measure of muscle mass or physical function, sarcopenia should be regarded as a plausible rather than a demonstrated mechanism underlying the observed association. A low GNRI may thus identify patients prone to recurrent ischaemia and progressive coronary disease, not only to fatal events.

Consistent with this rationale, GNRI has been used for risk assessment in heart failure, chronic kidney disease, haemodialysis, cardiac surgery, cardiac resynchronisation therapy, and PCI (13, 15, 36–42). Lower GNRI independently predicts adverse events in heart failure (15, 36), subsequent acute kidney injury in acute heart failure (37), postoperative infection and delirium in cardiac surgery (38, 39), and long-term outcomes after cardiac resynchronisation therapy (40). This evidence, however, comes largely from single-disease cohorts, and whether GNRI retains prognostic value within the broader CKM framework, particularly in CKM Stage 4 patients undergoing PCI, has remained insufficiently defined. Impaired nutrition may carry greater prognostic weight in CKM than in the general population. The conditions that define CKM, including chronic kidney disease with protein loss and uraemic anorexia, heart-failure cachexia, insulin resistance, and chronic inflammation, both raise the prevalence of malnutrition and worsen its prognostic impact (43); in patients with overlapping coronary and renal disease, malnutrition is highly prevalent and independently predicts mortality and major cardiovascular events (44). Our study addresses this gap by showing that GNRI was consistently associated with adverse outcomes across a population-based CKM cohort and an external high-risk PCI cohort, supporting its role as an integrated risk marker in CKM syndrome.

The present study combined population-based discovery with external validation in a clinically defined high-risk PCI cohort, allowing assessment of the consistency and generalisability of the GNRI–outcome association across distinct CKM settings. Despite substantial differences in population characteristics and clinical context, the prognostic signal of GNRI was remarkably consistent across both cohorts. The NHANES sample reflects the general US adult population spanning CKM Stages 1–4, with relatively low absolute event rates and modest comorbidity burden; the Beijing Luhe cohort represents a homogeneous ethnic population, a specific clinical context (post-PCI), and uniformly Stage 4 disease with high event rates (3-year MACE incidence >50% in Q1). Nevertheless, the qualitative direction and magnitude of the GNRI–outcome relationship are remarkably concordant, with both cohorts showing roughly a doubling of fully adjusted hazards in the lowest vs. highest GNRI quartile and risk thresholds near GNRI 100. This cross-cohort concordance is one of the most stringent forms of external validity, suggesting that the prognostic signal of GNRI is not driven by population-specific confounders or measurement artefacts.

Differences in subgroup patterns are also instructive. In NHANES, significant interactions emerged for sex and 10-year CVD risk score, possibly reflecting hormonal cardioprotection in pre-menopausal women (45), gender-related differences in lifestyle and body composition (46–48), or attenuation in patients already at very high baseline risk. In the validation cohort, the only significant effect modification was by glycaemic control (*P*-interaction = 0.012), with the prognostic effect of a low GNRI stronger in patients with HbA1c ≥ 6.5% than in those with better control; across the remaining strata the signal was directionally consistent despite clinical, physiological, and demographic heterogeneity, reinforcing the robustness of the marker. Poor glycaemic control promotes chronic inflammation, oxidative stress, advanced glycation, and endothelial dysfunction, which accelerate atherosclerosis (49). Superimposed on the inflammation and depleted reserve that a low GNRI reflects, these effects may compound one another, so that nutritional vulnerability and dysglycaemia together confer greater risk than either alone; the relationship is plausibly bidirectional, as malnutrition aggravates insulin resistance while dysglycaemia promotes protein catabolism. Consistent with this, a low GNRI has been reported to carry a more pronounced adverse prognostic impact in diabetic than in non-diabetic patients (50). In better-controlled patients, a less metabolically adverse phenotype, GNRI remained predictive but with an attenuated effect, suggesting that nutritional status is especially informative when metabolic stress is high.

Beyond demonstrating that GNRI was statistically associated with adverse outcomes, we showed that adding GNRI to a base clinical model—comprising age, sex, systolic blood pressure, BMI, LDL-C, eGFR, and HbA1c—improved Harrell's C-index for MACE by an absolute 0.048 (*P* < 0.001), exceeding the prevailing threshold of 0.02 considered clinically meaningful in cardiovascular risk-prediction modelling. Similar gains were observed for all-cause mortality and ischaemia-driven revascularisation, while time-dependent AUCs reached 0.645 at 3 years for MACE. Although the absolute discrimination values remain modest—reflecting the inherent biological heterogeneity of post-PCI outcomes in CKM Stage 4 patients—the magnitude of improvement attributable to a single, low-cost biomarker is substantial. Importantly, the model demonstrated good calibration, with all observed-vs.-predicted absolute deviations below 5% across quartile × time-point combinations.

Together, these findings suggest that GNRI may be incorporated into post-PCI risk-stratification workflows for patients with CKM Stage 4. Because GNRI requires only serum albumin and body weight, it can be calculated at admission using routinely available clinical data. GNRI is best regarded as an inexpensive, routinely derivable marker that complements established prognostic markers and clinical assessment rather than serving as a standalone risk-stratification tool. A low GNRI is a final common pathway rather than a single condition, and its cause should be identified before management is individualised. In CKM Stage 4 it may reflect cardiac cachexia (51), protein-energy wasting from chronic kidney disease (43), chronic systemic inflammation, occult malignancy, age-related sarcopenia (18, 33–35), or simply inadequate intake in a frail patient; in the cardiorenal setting these wasting processes frequently coexist (52) and carry different prognostic and therapeutic implications. A practical first step is to review dietary intake, weight trajectory, and functional status and to check inflammatory, renal, and cardiac indices; unexplained or disproportionate weight loss should prompt age-appropriate malignancy screening, and muscle mass or strength should be assessed where feasible. Because additional nutritional support alone may be insufficient when an ongoing catabolic process is present (51), the management measures outlined below should follow from, and be tailored to, this assessment. Accordingly, patients with GNRI < 100, particularly those in the lowest quartile, may warrant closer follow-up and intensified secondary prevention, together with cause-directed nutritional and multidisciplinary management involving dietetic support and cardiac rehabilitation. The consistent risk increase below GNRI ≈ 100 in both cohorts further supports this threshold as a practical candidate for clinical triage.

### 4.1 Limitations

Several limitations warrant acknowledgement. First, both analyses are observational, so causality cannot be established; residual confounding and reverse causation cannot be excluded, particularly in the discovery cohort, where a single baseline GNRI was followed over many years. Second, both GNRI components may be unstable in acute illness, as albumin reflects inflammatory and acute-phase physiology and body weight reflects volume status; pre-procedural admission values and cross-cohort consistency mitigate but do not eliminate this. Third, the validation cohort is single-centre, Chinese, and predominantly male (81%), so generalisability to women, in whom the subgroup was underpowered, remains uncertain. Fourth, its median follow-up of 1.58 years limits very long-term inference. Fifth, body-composition assessment was limited: waist circumference and waist-to-height ratio were unavailable, the BMI-22 anchor may fit ethnic groups unequally, and no direct measure of muscle mass was available, so sarcopenia remains only a plausible contributor. Sixth, several variables were uncaptured in the validation cohort, including peri-procedural markers such as NT-proBNP and hs-CRP and secondary-prevention medications, leaving residual confounding by differential treatment. Finally, the two-cohort design is best read as convergent evidence rather than formal validation, since quartiles, covariates, and outcomes differed, and the Stage 4 findings should not be uncritically extrapolated to Stages 1–3.

## 5 Conclusions

In this discovery–validation study, lower GNRI was independently and dose-dependently associated with adverse outcomes across the CKM continuum in a nationally representative NHANES cohort, and these findings were externally validated in 2,401 patients with CKM Stage 4 undergoing PCI. Low GNRI was robustly associated with MACE, all-cause death, and ischaemia-driven repeat revascularisation, and adding GNRI to a comprehensive clinical model improved risk discrimination and calibration across these distinct CKM settings. Because GNRI can be derived from routinely available data, admission assessment, particularly of values near or below 100, may help flag higher-risk patients for closer follow-up and nutritional evaluation. Whether GNRI-guided management can improve outcomes remains uncertain and warrants prospective investigation.

## Data Availability

Publicly available datasets were analyzed in this study. This data can be found here: the U.S. National Health and Nutrition Examination Survey (NHANES) database (https://wwwn.cdc.gov/nchs/nhanes/).
