# Supplementary material for: Geriatric nutritional risk Index predicts adverse outcomes across cardiovascular–kidney–metabolic syndrome: discovery in NHANES and external validation in CKM stage 4 patients undergoing PCI
Source: Front Cardiovasc Med. 2026 Jul 7;13:1878842. doi: 10.3389/fcvm.2026.1878842 (PMC13385701; doi:10.3389/fcvm.2026.1878842)
Supplement: Supplementary file 1 [file Datasheet1.pdf]

Supplementary Material

1 Supplementary Figures and Tables

1.1 Supplementary Figures

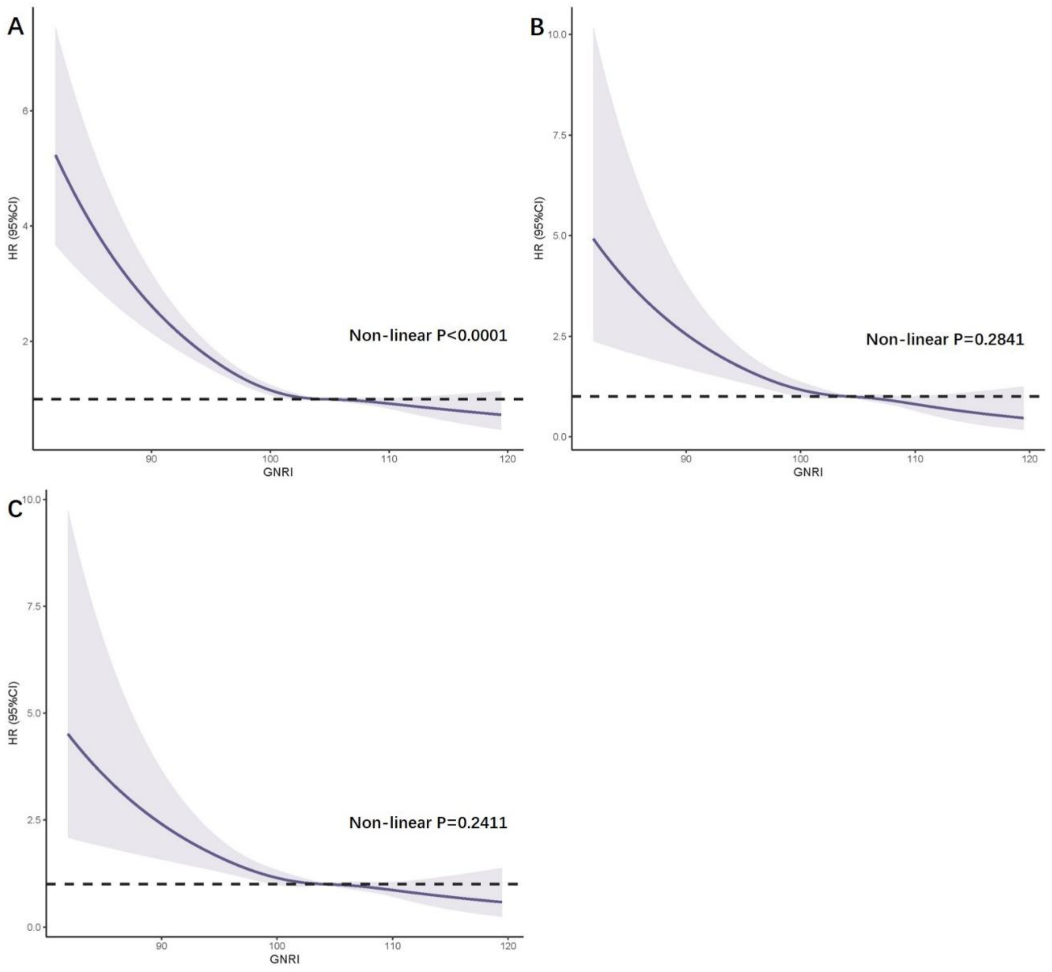

Supplementary Figure S1. The figure legends are required to have the same font as the main text, 12 point normal Times New Roman, single spaced. Please use a single paragraph for each legend and prepare the figures keeping in mind the PDF layout.

Supplementary Figure S1. Restricted cubic spline (RCS) curves illustrating the dose–response relationships between GNRI and mortality outcomes in the discovery cohort: (A) all-cause mortality, (B) cardiovascular mortality, and (C) cancer mortality. The shaded area represents the 95% confidence interval. A non-linear inverse relationship was observed for all-cause mortality (P-non-linear < 0.0001), with the steepest risk increase below GNRI ≈ 100. The associations with

cardiovascular and cancer mortality were monotonic but did not reach formal evidence of non-linearity (P-non-linear = 0.284 and 0.241, respectively).

Abbreviations: CVD, cardiovascular disease; GNRI, Geriatric Nutritional Risk Index; HR, hazard ratio; RCS, restricted cubic spline.

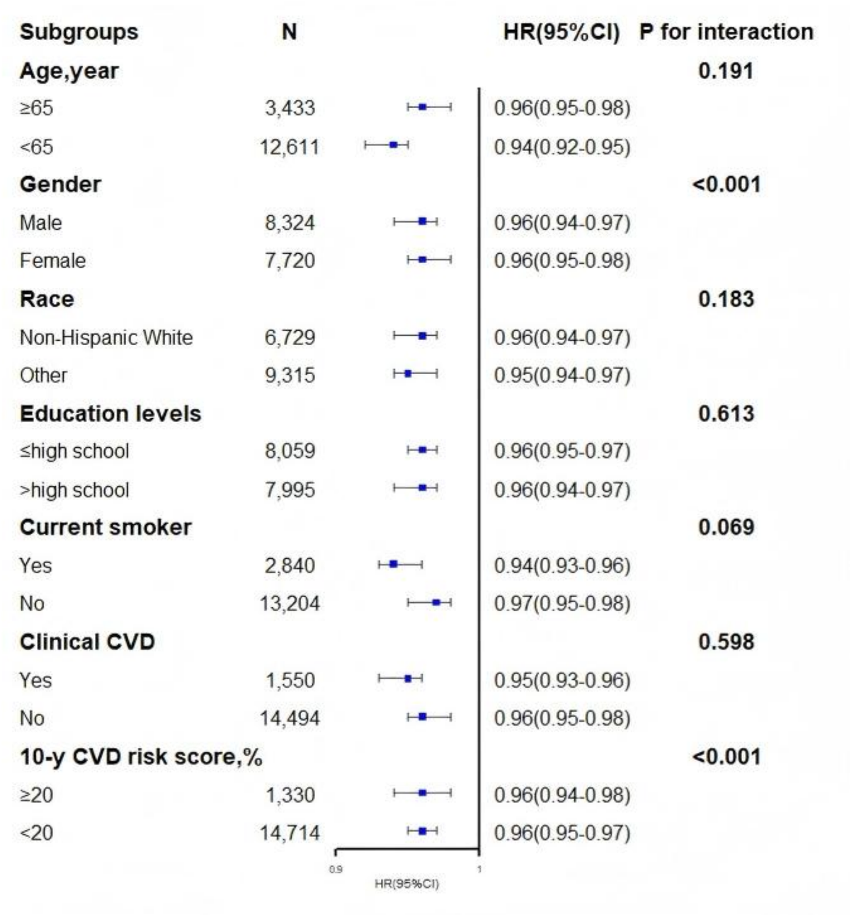

Supplementary Figure S2. Forest plot of subgroup analyses showing the association between GNRI and all-cause mortality in the **discovery** cohort, with hazard ratios (HRs) and 95% confidence intervals (CIs) presented across subgroups defined by age, sex, race, education level, smoking status, clinical CVD history, and 10-year CVD risk score. Significant interactions were observed for sex and 10-year CVD risk score (both P-interaction < 0.001).

Abbreviations: CI, confidence interval; CVD, cardiovascular disease; GNRI, Geriatric Nutritional Risk Index; HR, hazard ratio.

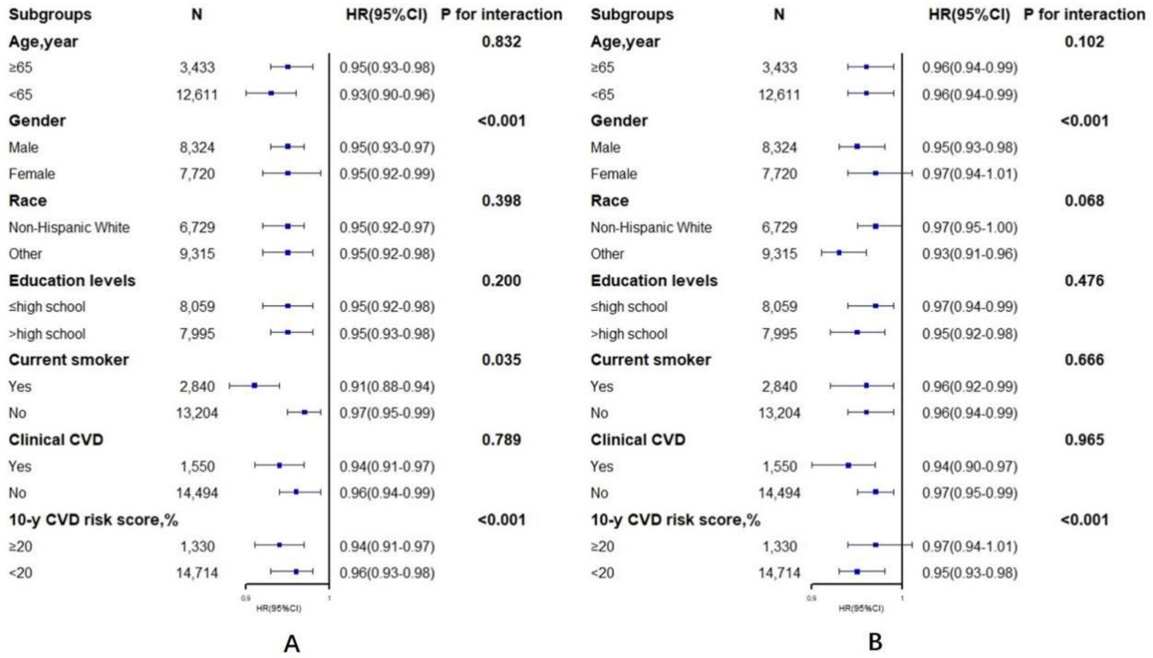

Supplementary Figure S3. Subgroup analyses of the association between GNRI and cause-specific mortality in the discovery cohort, showing (A) cardiovascular mortality and (B) cancer mortality. Hazard ratios and 95% confidence intervals are presented across subgroups defined by age, sex, race, education level, smoking status, clinical CVD history, and 10-year CVD risk score. Significant sex and 10-year CVD risk interactions were detected for both endpoints.

Abbreviations: CI, confidence interval; CVD, cardiovascular disease; GNRI, Geriatric Nutritional Risk Index; HR, hazard ratio.

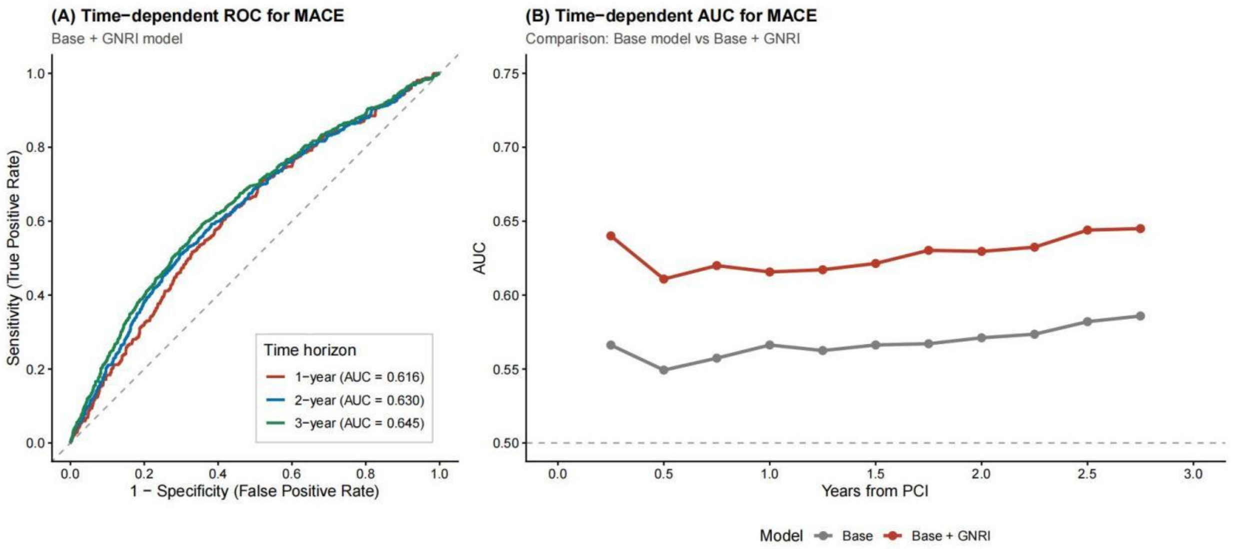

Supplementary Figure S4. Time-dependent receiver operating characteristic (ROC) analysis for MACE in the external validation cohort. (A) Time-dependent ROC curves of the base + GNRI model

at 1, 2, and 3 years (AUCs of 0.616, 0.630, and 0.645, respectively). (B) Time-dependent area under the curve (AUC) trajectories comparing the base model with the base + GNRI model across the entire follow-up window; the GNRI-augmented model uniformly outperformed the base model.

Abbreviations: AUC, area under the curve; GNRI, Geriatric Nutritional Risk Index; MACE, major adverse cardiovascular event; PCI, percutaneous coronary intervention; ROC, receiver operating characteristic.

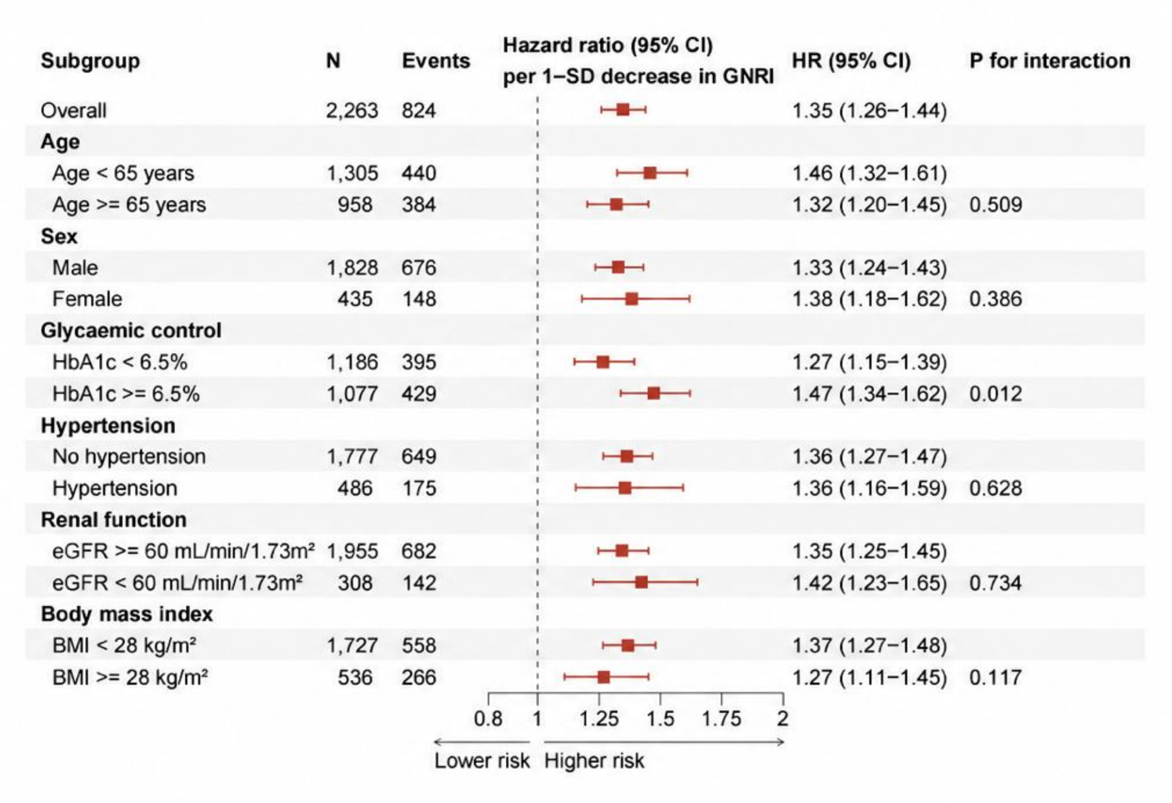

Supplementary Figure S5. Forest plot of subgroup analyses for the association between GNRI and MACE in the external validation cohort (n = 2,263). Hazard ratios are expressed per 1-SD decrease in GNRI, with 95% confidence intervals shown across subgroups defined by age, sex, diabetes status (HbA1c < 6.5% vs ≥ 6.5%), hypertension, renal function (eGFR ≥ 60 vs < 60 mL/min/1.73 m²), and BMI (< 28 vs ≥ 28 kg/m²). The overall hazard ratio per 1-SD decrement in GNRI was 1.35 (95% CI 1.26–1.44), with directionally consistent associations across all subgroups; a statistically significant interaction was detected for glycaemic control (HR 1.27 in HbA1c < 6.5% vs HR 1.47 in HbA1c ≥

6.5%; P-interaction = 0.012), indicating a stronger prognostic effect of GNRI in patients with poorer glycaemic control.

Abbreviations: BMI, body mass index; CI, confidence interval; eGFR, estimated glomerular filtration rate; GNRI, Geriatric Nutritional Risk Index; HbA1c, glycated haemoglobin; HR, hazard ratio; MACE, major adverse cardiovascular event; SD, standard deviation.

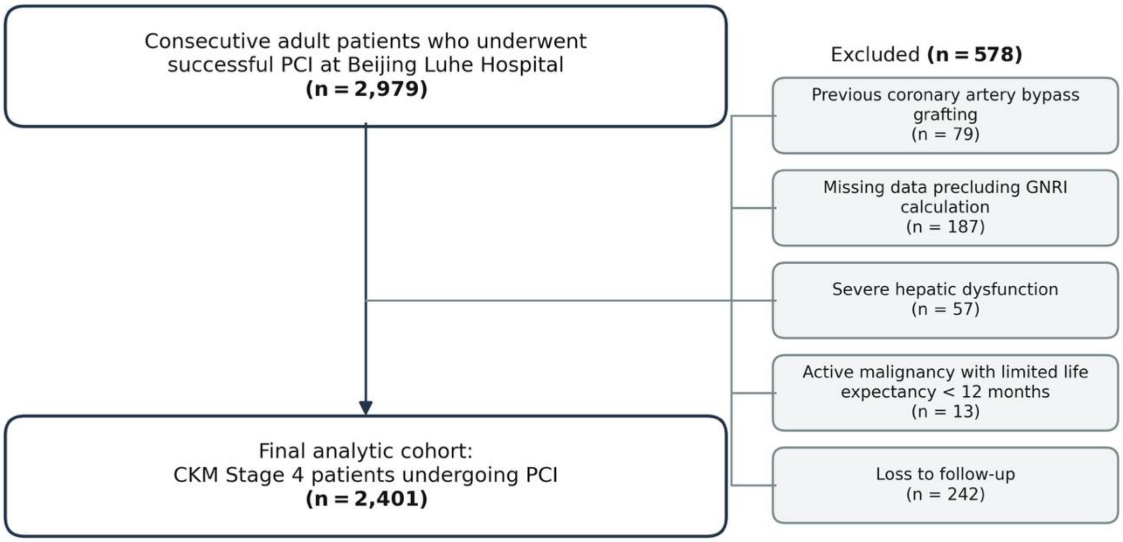

Supplementary Figure S6. Participant-selection flow diagram for the external validation cohort. Of 2,979 consecutive adult patients who underwent successful percutaneous coronary intervention (PCI) at Beijing Luhe Hospital, 578 were excluded for a history of coronary artery bypass grafting (n = 79), missing data precluding GNRI calculation (n = 187), severe hepatic dysfunction (n = 57), or active malignancy with limited life expectancy < 12 months (n = 13), as well as loss to follow-up (n = 242), leaving 2,401 patients with cardiovascular–kidney–metabolic (CKM) Stage 4 for analysis.

Abbreviations: GNRI, Geriatric Nutritional Risk Index; MACE, major adverse cardiovascular events; PCI, percutaneous coronary intervention.

1.2 Supplementary Tables

Supplementary Table S1. Mortality outcomes in the discovery cohort (NHANES 1999–2018) stratified by GNRI quartiles.

Outcome	Overall (N = 16,074)	Quartiles of GNRI				P value
		Q1 (N = 4,894)	Q2 (N = 3,763)	Q3 (N = 3,710)	Q4 (N = 3,707)	
All-cause mortality	1,912 (9.4)	680 (11.3)	477 (10.5)	423 (8.7)	332 (6.9)	<0.001
CVD mortality	475 (2.3)	184 (3.3)	109 (2.3)	107 (2.1)	75 (1.5)	<0.001
Cancer mortality	515 (2.5)	164 (2.6)	148 (3.0)	121 (2.5)	82 (1.9)	0.067

Values are n (weighted %). P values from the χ^2 test.

Abbreviations: CVD, cardiovascular disease; GNRI, Geriatric Nutritional Risk Index.

Supplementary Table S2. Cox proportional hazards models for the association between GNRI and mortality outcomes in the discovery cohort.

GNRI category	Model 1 (Unadjusted)		Model 2 (Age, sex, race)		Model 3 (Fully adjusted)	
	HR (95% CI)	P	HR (95% CI)	P	HR (95% CI)	P
All-cause mortality (P for trend < 0.001 in all models)						
Q4 (highest, reference)	1.00 (Ref)	—	1.00 (Ref)	—	1.00 (Ref)	—
Q3	1.42 (1.17–1.71)	<0.001	1.19 (1.00–1.42)	0.050	1.14 (0.95–1.37)	0.147
Q2	1.79 (1.48–2.18)	<0.001	1.38 (1.16–1.65)	<0.001	1.31 (1.09–1.57)	0.004
Q1 (lowest)	2.30 (1.97–2.69)	<0.001	1.94 (1.64–2.30)	<0.001	1.58 (1.32–1.89)	<0.001
GNRI per 1-unit increase	0.93 (0.92–0.94)	<0.001	0.93 (0.92–0.95)	<0.001	0.95 (0.94–0.97)	<0.001
Cardiovascular mortality (P for trend < 0.001 in all models)						
Q4 (highest, reference)	1.00 (Ref)	—	1.00 (Ref)	—	1.00 (Ref)	—
Q3	1.60 (1.09–2.33)	0.016	1.37 (0.95–1.96)	0.091	1.25 (0.87–1.81)	0.225
Q2	1.86 (1.27–2.72)	0.001	1.50 (1.05–2.15)	0.028	1.35 (0.93–1.96)	0.115
Q1 (lowest)	3.20 (2.33–4.41)	<0.001	2.91 (2.13–3.98)	<0.001	2.13 (1.55–2.93)	<0.001
GNRI per 1-unit increase	0.91 (0.89–0.93)	<0.001	0.91 (0.89–0.93)	<0.001	0.94 (0.91–0.96)	<0.001
Cancer mortality (P for trend = 0.004 / <0.001 / <0.001 across models)						
Q4 (highest, reference)	1.00 (Ref)	—	1.00 (Ref)	—	1.00 (Ref)	—
Q3	1.43 (1.02–2.01)	0.036	1.20 (0.85–1.71)	0.291	1.18 (0.83–1.67)	0.346
Q2	1.81 (1.27–2.59)	0.001	1.40 (0.99–1.97)	0.056	1.37 (0.97–1.92)	0.071
Q1 (lowest)	1.87 (1.31–2.68)	0.001	1.59 (1.10–2.28)	0.013	1.41 (0.98–2.02)	0.066
GNRI per 1-unit increase	0.95 (0.92–0.97)	<0.001	0.96 (0.93–0.98)	0.001	0.96 (0.94–0.99)	0.013

Hazard ratios (95% confidence intervals) are derived from Cox proportional hazards models. Model 1: unadjusted. Model 2: adjusted for age, sex, and race. Model 3: fully adjusted for age, sex, race, education, current smoking, systolic blood pressure, waist circumference, low-density lipoprotein cholesterol, estimated glomerular filtration rate, and urine albumin-to-creatinine ratio. The "GNRI per 1-unit increase" row reports the hazard ratio for each 1-point increment in the continuous GNRI score; HRs < 1 indicate that higher GNRI (better nutritional status) is associated with lower mortality.

Abbreviations: CI, confidence interval; CVD, cardiovascular disease; GNRI, Geriatric Nutritional Risk Index; HR, hazard ratio.

Supplementary Table S3. Incidence of cardiovascular outcomes by GNRI quartile in patients with CKM Stage 4 undergoing PCI (N = 2,401).

Outcome	Q1 (n = 611)	Q2 (n = 598)	Q3 (n = 602)	Q4 (n = 590)	Total (n = 2,401)	P value
MACE (composite)						
Events, n (%)	313 (51.2)	238 (39.8)	191 (31.7)	138 (23.4)	880 (36.7)	<0.001

Person-years of follow-up	1,310.5	1,386.2	1,464.9	1,550.3	5,711.8	—
Incidence rate per 100 PY (95% CI)	23.88 (21.38–26.68)	17.17 (15.12–19.50)	13.04 (11.31–15.02)	8.90 (7.53–10.52)	15.41 (14.42–16.46)	—
Cumulative incidence at 1 year, %	22.3	18.4	15.8	9.5	—	—
Cumulative incidence at 2 years, %	40.1	31.1	25.9	18.6	—	—
Cumulative incidence at 3 years, %	51.2	39.8	31.7	23.4	—	—
All-cause death						
Events, n (%)	152 (24.9)	119 (19.9)	87 (14.5)	66 (11.2)	424 (17.7)	<0.001
Incidence rate per 100 PY (95% CI)	11.60 (9.89–13.60)	8.58 (7.17–10.27)	5.94 (4.81–7.33)	4.26 (3.34–5.42)	7.42 (6.75–8.16)	—
Cumulative incidence at 1 year, %	11.4	9.8	7.6	4.3	—	—
Cumulative incidence at 2 years, %	22.3	16.9	12.6	9.5	—	—
Cumulative incidence at 3 years, %	29.4	22.3	15.8	12.0	—	—
Non-fatal myocardial infarction						
Events, n (%)	28 (4.6)	24 (4.0)	24 (4.0)	14 (2.4)	90 (3.7)	0.064
Incidence rate per 100 PY (95% CI)	2.14 (1.48–3.09)	1.73 (1.16–2.58)	1.64 (1.10–2.44)	0.90 (0.53–1.52)	1.58 (1.28–1.94)	—
Cumulative incidence at 1 year, %	2.3	1.4	1.4	1.2	—	—
Cumulative incidence at 2 years, %	4.3	3.2	3.0	2.0	—	—
Cumulative incidence at 3 years, %	6.0	5.2	4.8	2.6	—	—
Ischaemia-driven repeat revascularisation						
Events, n (%)	133 (21.8)	95 (15.9)	80 (13.3)	58 (9.8)	366 (15.2)	<0.001
Incidence rate per 100 PY (95% CI)	10.15 (8.56–12.03)	6.85 (5.61–8.38)	5.46 (4.39–6.80)	3.74 (2.89–4.84)	6.41 (5.78–7.10)	—
Cumulative incidence at 1 year, %	10.0	8.1	7.5	4.2	—	—
Cumulative incidence at 2 years, %	19.1	14.2	12.4	8.2	—	—
Cumulative incidence at 3	26.2	18.1	14.7	10.5	—	—

years, %

MACE was defined as a composite of all-cause death, non-fatal myocardial infarction, and ischaemia-driven repeat revascularisation. Events n (%) represent crude proportions of patients in each quartile experiencing the outcome during follow-up; the corresponding P value is from Pearson's χ^2 test (concordant with the log-rank P shown for completeness). Incidence rates are expressed per 100 person-years with 95% confidence intervals. Cumulative incidence was estimated as 1 minus the Kaplan–Meier survivor function. "—" denotes not applicable for the pooled column. P values for the comparison across GNRI quartiles are derived from the log-rank test. Median follow-up time was 1.58 years (interquartile range, 0.69 to 3.00 years); total observation = 5,711.8 person-years.

Abbreviations: CI, confidence interval; CKM, cardiovascular–kidney–metabolic; GNRI, Geriatric Nutritional Risk Index; MACE, major adverse cardiovascular event; PCI, percutaneous coronary intervention; PY, person-years; Q, quartile.

Supplementary Table S4. Calibration of the base + GNRI Cox model for MACE at 1, 2, and 3 years in the validation cohort.

Time horizon	Risk group	Predicted MACE risk (%)	Observed MACE risk, % (95% CI)	Difference, % (Obs − Pred)
1-year	Q1 (lowest)	10.2	9.7 (7.0–12.4)	−0.5
	Q2	13.5	13.1 (9.9–16.3)	−0.5
	Q3	16.5	18.6 (14.6–22.5)	+2.0
	Q4 (highest)	23.9	23.1 (18.6–27.7)	−0.7
2-year	Q1 (lowest)	18.9	18.7 (14.8–22.7)	−0.2
	Q2	24.7	23.7 (19.1–28.3)	−1.0
	Q3	29.7	29.0 (23.7–34.2)	−0.7
	Q4 (highest)	41.0	43.5 (36.2–50.7)	+2.5
3-year	Q1 (lowest)	24.6	23.0 (18.5–27.5)	−1.6
	Q2	31.7	29.7 (24.3–35.0)	−2.1
	Q3	37.8	38.3 (31.8–44.8)	+0.6
	Q4 (highest)	50.5	54.1 (45.1–63.0)	+3.5

Patients (n = 2,264) were stratified into quartiles (Q1–Q4) of predicted MACE risk based on the linear predictor of the base + GNRI Cox model (covariates: age, sex, systolic blood pressure, body mass index, low-density lipoprotein cholesterol, estimated glomerular filtration rate, glycated haemoglobin, and GNRI). Predicted MACE risk represents the mean within-quartile model-derived event probability at the corresponding time horizon $(1 - e^{(-H(t))})$. Observed MACE risk is the within-quartile event rate estimated by the Kaplan–Meier method, with 95% CI derived from 1,000 bootstrap replications. All absolute deviations between observed and predicted risks were below 5% across the 12 quartile × time-point combinations. The 95% CIs of all observed estimates encompassed the corresponding predicted values, supporting good calibration of the base + GNRI model in this external cohort.

Abbreviations: CI, confidence interval; GNRI, Geriatric Nutritional Risk Index; MACE, major adverse cardiovascular event; Obs, observed; Pred, predicted; Q, quartile.
